# Beta-ketothiolase deficiency with progressive basal ganglia and extra basal ganglia involvement: CT–MRI correlation in a pediatric metabolic encephalopathy: A case report

**DOI:** 10.1016/j.radcr.2026.06.125

**Published:** 2026-07-22

**Authors:** Berihu Gebre, Yirgalem Gereziher, Dejen Gebrewahd, Catherine Alcober

**Affiliations:** a Department of Radiology, Nauru Medical Center, Republic of Nauru; b Department of Pediatric Surgery, Mekelle University, Mekele, Ethiopia; c Department of Neurosurgery, Mekelle University, Mekele, Ethiopia; d Department of Pediatric, Nauru Medical Center, Republic of Nauru

**Keywords:** Beta-ketothiolase deficiency, BKTD, Basal ganglia, Globus pallidus, Cerebral peduncle, Inborn error of metabolism, SWI

## Abstract

Beta-ketothiolase deficiency (BKTD), also called mitochondrial acetoacetyl-CoA thiolase (T2) deficiency, is a rare autosomal recessive inborn error of metabolism affecting isoleucine catabolism and ketone body utilization. Although recurrent ketoacidotic crises are the hallmark of the disease, neurological complications—particularly basal ganglia injury—are increasingly recognized. We report a 2-year-old girl with known BKTD who presented with severe euglycemic ketoacidosis and acute encephalopathy. Initial CT showed symmetric hypodensity confined to the bilateral globus pallidi. Follow-up CT during ongoing metabolic instability demonstrated interval progression to involve the bilateral putamina and cerebral peduncles. MRI, obtained after referral, revealed nonenhancing T2/FLAIR hyperintense, T1 hypointense globus pallidus lesions without diffusion restriction but with punctate SWI hypointensities consistent with microcystic cavitary degeneration and microhemorrhage. There were additional diffusion-restricting lesions in the bilateral cerebral peduncles, small nonrestricting white matter foci in the frontal and right parietal lobes, and generalized cerebral atrophy. A baseline MRI 17 months earlier had been normal. This case illustrates the evolution from acute pallidal injury to irreversible basal ganglia necrosis with microhemorrhage and concurrent acute/subacute tract involvement, and highlights how CT and MRI together can characterize the spectrum of BKTD-related brain injury.

## Introduction

Beta-ketothiolase deficiency (BKTD), or mitochondrial acetoacetyl-CoA thiolase (T2) deficiency, is a rare autosomal recessive disorder of isoleucine and ketone body metabolism caused by mutations in ACAT1 [1]. Defective thiolytic cleavage of 2-methylacetoacetyl-CoA impairs ketone utilization and isoleucine catabolism, leading to accumulation of organic acids (e.g., 2-methyl-3-hydroxybutyrate, 2-methylacetoacetate, and tiglylglycine) and episodic severe ketoacidosis [1]. Clinically, BKTD typically presents in infancy or early childhood with vomiting, dehydration, Kussmaul respirations, lethargy, seizures, and coma, often precipitated by infection or fasting [[1], [2], [3]].

Neuroimaging changes are increasingly recognized in BKTD and may manifest as toxic–metabolic encephalopathy or “metabolic stroke,” most commonly affecting the basal ganglia [[1], [2], [3]]. CT and MRI typically demonstrate bilateral abnormalities in the globus pallidus and/or putamina, with variable extension to white matter, brainstem, and cerebellum [[1], [2], [3], [4], [5], [6]].

We present a pediatric case of BKTD with progressive CT changes and characteristic MRI findings involving the globus pallidi, cerebral peduncles, and supratentorial white matter. This case highlights the value of serial CT when MRI is limited and illustrates the evolution from acute metabolic injury to irreversible basal ganglia damage and tract involvement.

## Case report

A 2-year-old Nauruan girl with known beta-ketothiolase (T2) deficiency presented to our institution with acute metabolic decompensation and encephalopathy.

### Prior history and initial diagnosis

Seventeen months before the current admission, at the age of approximately 7 months, the patient had been hospitalized at an overseas center with an episode of depressed consciousness following an upper respiratory tract infection. At that time, she was noted to have tachypnea and decreased responsiveness. Laboratory evaluation revealed severe metabolic acidosis with markedly elevated anion gap and strong ketonuria; lactate was not significantly elevated. An inborn error of metabolism was suspected.

Because of concern for infectious or inflammatory encephalitis or demyelinating disease, a brain MRI was obtained. That MRI demonstrated normal brain parenchyma, without abnormal T2/FLAIR hyperintensity, no restricted diffusion, and no abnormal enhancement. There was no evidence of encephalitis, demyelination, or a primary metabolic leukoencephalopathy. No basal ganglia, brainstem, or cerebellar signal abnormalities were identified at that time (Fig. 1A-D).

**Fig. 1:**
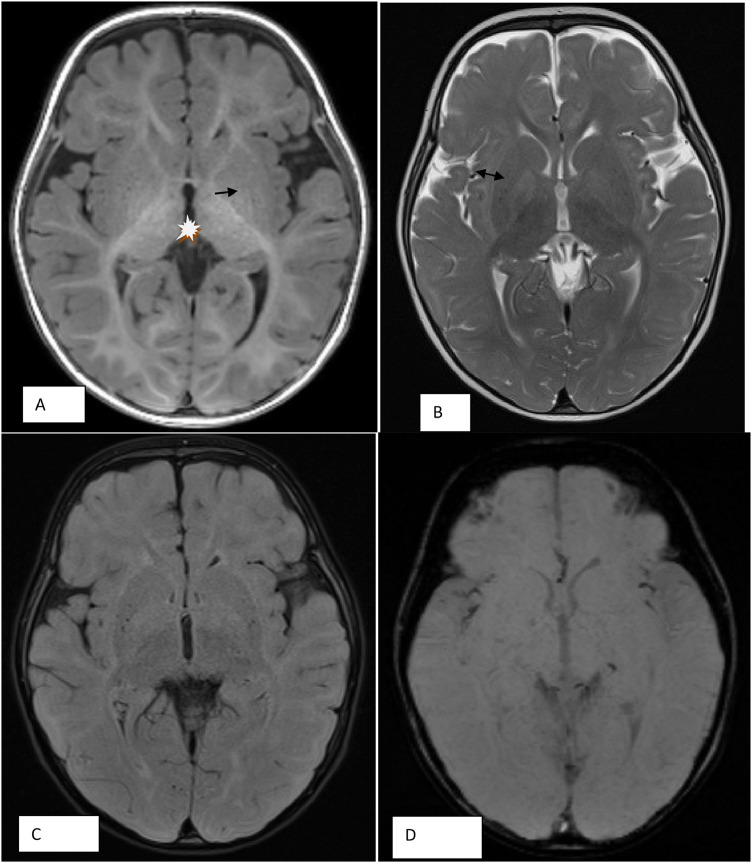
Axial T1-weighted (A), T2-weighted (B), FLAIR (C), and SWI (D) images at the level of the third ventricle demonstrate normal signal intensity of the cerebral white matter and gray matter. No abnormal signal intensity or susceptibility is identified. The black single arrow (→) indicates the normal globus pallidus, and the black double-headed arrow (⟷) indicates the lentiform nucleus.

Given the unexplained severe ketoacidosis and normal structural MRI, a comprehensive metabolic and genetic evaluation was pursued. Urine organic acid analysis showed a pattern consistent with an organic acidemia; subsequent extended genetic testing identified homozygosity for the I323V mutation in the acetyl-CoA acetyltransferase 1 (ACAT1) gene. This I323V variant has been well established as a pathogenic mutation causing beta-ketothiolase deficiency [2]. Based on the biochemical and molecular findings, a diagnosis of beta-ketothiolase deficiency was made. The patient was started on dietary management, including avoidance of prolonged fasting and moderate protein restriction, together with carnitine supplementation, and she remained clinically stable until the current episode.

### Current presentation

At 2 years of age, the patient was admitted to our hospital with a 1-day history of vomiting and diarrhea, leading to moderate dehydration. Initially, she was alert but irritable. Intravenous fluids were administered, and gastrointestinal symptoms improved.

Within 12 hours of admission, she developed progressive respiratory distress with wheezing and increased work of breathing. On hospital day 3, she acutely deteriorated neurologically: her Glasgow Coma Scale score fell to 8 (E2V2M4), with poor eye opening, incomprehensible sounds, and withdrawal to pain only. She was tachypneic and tachycardic, with borderline hypotension and persistent signs of dehydration. Laboratory evaluation at this time revealed: Severe euglycemic metabolic ketoacidosis on central venous blood gas: pH 6.91, bicarbonate 3.4 mmol/L, and base excess −29. Strongly positive urine ketones. Lactate within normal limits. Leukocytosis and thrombocytosis, with preserved renal function (Fig. 2).

**Fig. 2:**
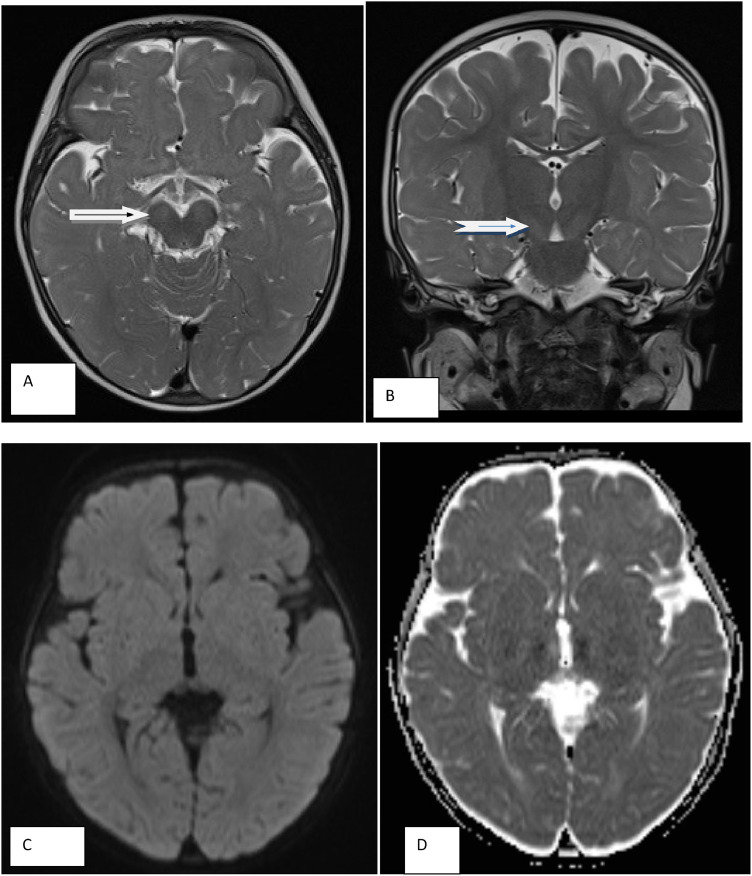
Axial T2-weighted (A) image at the level of the cerebral peduncles and coronal T2-weighted (B) image demonstrate normal signal intensity, with no evidence of demyelination or metabolic-related white or gray matter abnormalities. DWI (C) and ADC (D) maps show no diffusion restriction. The arrow with a black center indicates the cerebral peduncle, and the arrow with a blue center indicates the white matter tract.

These findings were consistent with an acute metabolic crisis due to BKTD, triggered by intercurrent gastrointestinal illness.

Subsequently CT scan was done to rule out metabolic related Brian disease and brain edema. Noncontrast CT of the brain (Fig. 3A-D) demonstrated bilateral, symmetric hypodensity of the globus pallidus, with mean attenuation of approximately 20 HU and no evidence of hemorrhage. In the setting of severe metabolic acidosis, this appearance was considered suggestive of early microcystic degeneration secondary to metabolic injury. A small additional hypodense focus was noted in the right parietal white matter, while the remainder of the brain parenchyma was unremarkable. There was no mass effect, midline shift, generalized cerebral edema, or hydrocephalus.

**Fig. 3:**
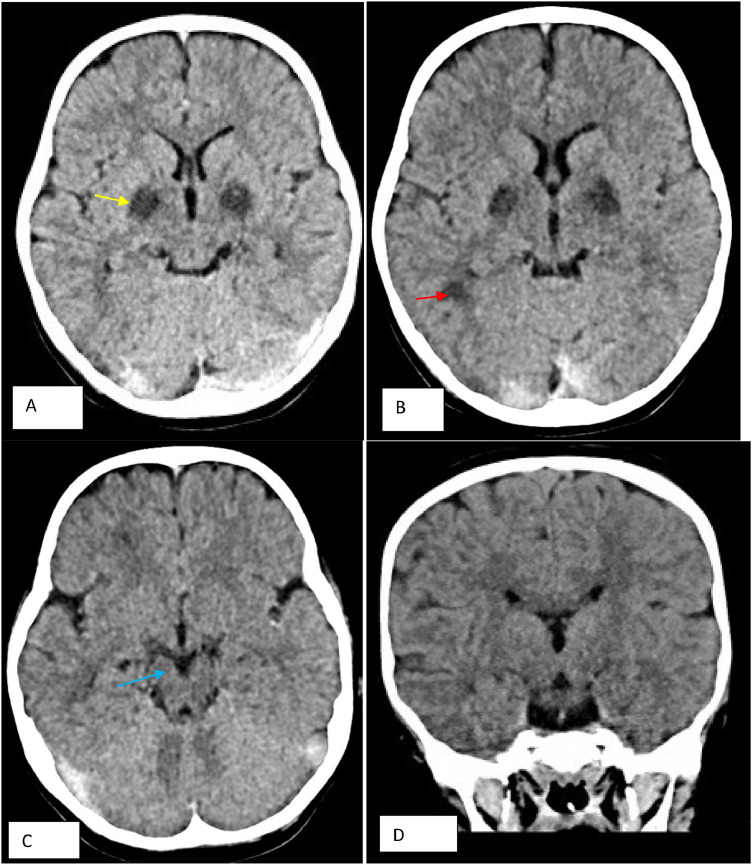
Axial (A-C) and coronal (D) representative images from the initial CT scan demonstrate bilateral, symmetric hypodensity of the globus pallidus, with a mean attenuation of approximately 20 HU and no evidence of hemorrhage (yellow arrow). In the setting of severe metabolic acidosis, this appearance was considered suggestive of early microcystic degeneration secondary to metabolic injury. A small additional hypodense focus is noted in the right parietal white matter (red arrow), while the remainder of the brain parenchyma is unremarkable. Blue arrow (cerebral peduncle).

Because of ongoing clinical deterioration, a follow-up non-contrast CT was obtained during continued metabolic instability (Fig. 4A-D). This study showed clear interval progression, with extension of the hypodensity from globipallidi to involve both putamina, resulting in more extensive lentiform nucleus involvement. In addition, new, symmetric hypodensities were identified in the bilateral cerebral peduncles and asymmetric hypodensity in the bilateral frontal lobe. This progressive, highly symmetric pattern indicated that the metabolic insult was not static and suggested that deep gray injury could propagate along myelinated white-matter pathways when metabolic derangement persisted. After correction of the metabolic acidosis, the patient’s level of consciousness improved and laboratory parameters normalized, and she was referred overseas for MRI and further management.

**Fig. 4:**
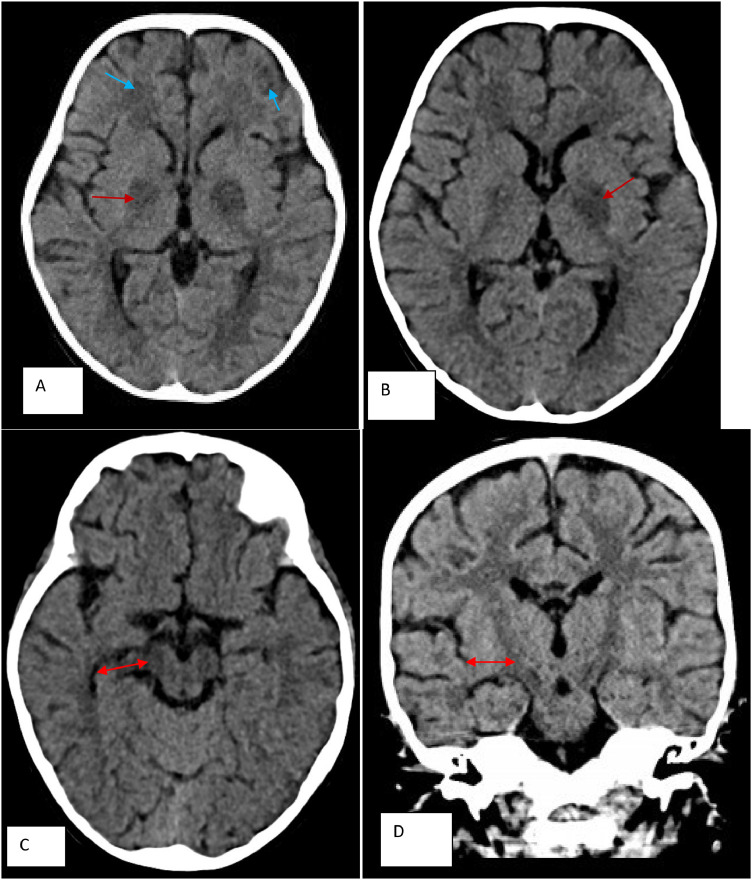
Repeat CT scan performed 6 days after the initial study shows axial (A-C) and coronal (D) images with clear interval progression. The hypodensity previously confined to the globus pallidus has extended to involve both putamina (single-headed red arrow), resulting in more extensive lentiform nucleus involvement. In addition, new, symmetric hypodensities are identified in the bilateral cerebral peduncles and white matter tracts (double-headed red arrow). Bilateral frontal white and gray matter hypodensities are also present (blue arrow). These new findings suggest extra–basal ganglia metabolic injury related to beta-ketothiolase deficiency.

Subsequent MRI demonstrated corresponding chronic and acute changes. Within the basal ganglia, the bilateral globus pallidi were T2- and FLAIR-hyperintense and T1-hypointense. There was no diffusion restriction in the globipallidi on DWI/ADC maps. Susceptibility-weighted imaging (SWI) revealed punctate foci of hypointensity within both globipallidi, compatible with microhemorrhages (Fig. 5A-F). Taken together, these features were interpreted as microcystic cavitary degeneration with associated microhemorrhage, representing irreversible basal ganglia injury and closely resembling the chronic stage described in prior BKTD reports [2,4]. In contrast, the bilateral cerebral peduncles and adjacent brainstem showed T2-hyperintense, T1-hypointense signal with corresponding diffusion restriction (DWI hyperintensity and low ADC) (Fig. 6A-D), in keeping with acute/subacute tract injury temporally related to the recent metabolic crisis. Additional small, scattered T2/FLAIR hyperintense foci were present in the subcortical and deep white matter of the bilateral frontal lobes and the right parietal lobe and temporal lobe, more prominent on the right, without diffusion restriction, suggesting subacute–chronic gliotic white-matter changes rather than acute infarction (Fig. 7A-D). Finally, there was generalized decrease in brain volume, with prominence of the ventricles and cortical sulci, compatible with diffuse cerebral atrophy (Fig. 6, Fig. 7A-D). Together, the CT and MRI findings support a pattern of irreversible pallidal degeneration with microhemorrhage, superimposed on acute/subacute peduncular tract injury and more diffuse white-matter and global brain involvement following severe metabolic decompensation in beta-ketothiolase deficiency.

**Fig. 5:**
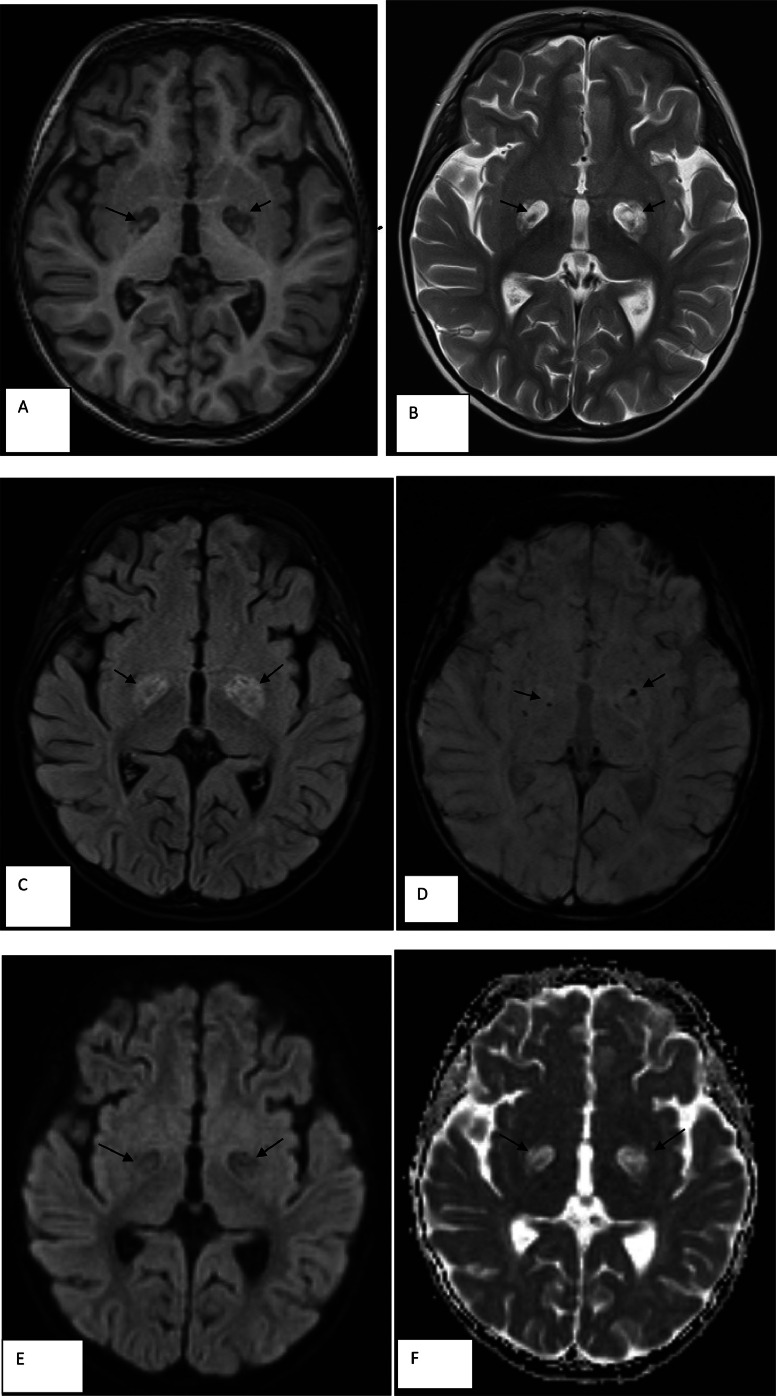
Axial T1W (A), T2W(B), FLAIR (C), and SWI (D) images at the level of the third ventricle demonstrate bilateral T1 hypointense and T2 hyperintense signal abnormalities in the globus pallidi, which are not suppressed on FLAIR. These lesions show internal susceptibility on SWI, consistent with microhemorrhages. There is no diffusion restriction on DWI (E) on ADC (F) mapping . Overall, the findings indicate symmetric microcystic changes of the bilateral globus pallidi (black arrow) with internal microhemorrhages in a patient with known beta-ketothiolase deficiency. The black arrow indicates the finding.

**Fig. 6:**
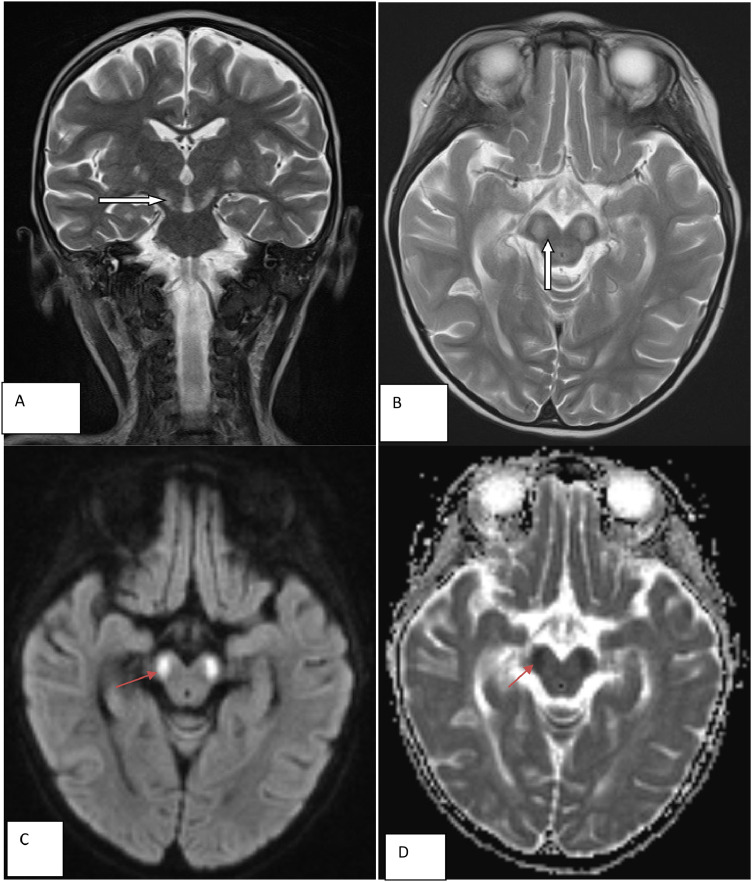
Coronal T2-weighted (A) and axial T2-weighted (B) images demonstrate generalized cerebral atrophy with T2 hyperintense signal abnormalities involving the bilateral cerebral peduncles and supratentorial white matter tracts. These areas show diffusion restriction on DWI and ADC mapping (high signal on DWI (C) and low signal on ADC (D)) in keeping with acute to subacute injury. In the context of known beta-ketothiolase deficiency, these findings indicate metabolic injury extending beyond the basal ganglia, with chronic changes in the globus pallidi and superimposed acute/subacute extra-basal ganglia involvement of the cerebral white matter and brainstem tracts. The black arrow indicate white matter tract (coronal image) and T2 (axial) hyper intense lesion restriction at the level of cerebral peduncle (red arrow).

**Fig. 7:**
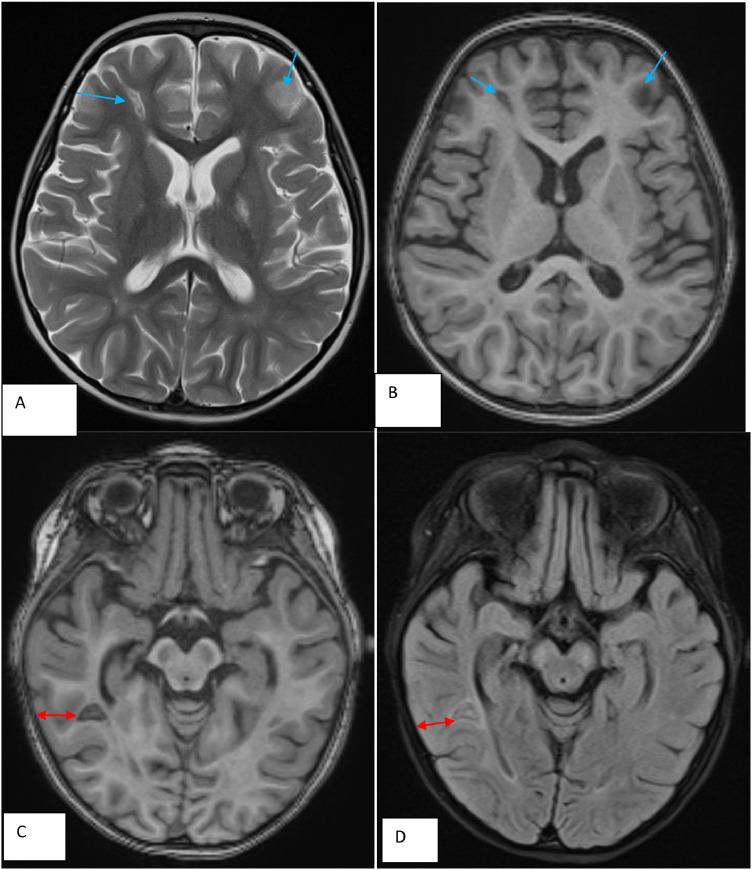
Axial T2 (A), T1 (B and C), FLIAR (D) image showed scattered T2/FLAIR hyperintense and T1W hypo intense foci in the subcortical and deep white matter of the bilateral frontal lobes, the right parietal lobe and temporal lobe, more prominent on the right without diffusion restriction, suggesting subacute–chronic gliotic white-matter changes rather than acute infarction related to beta-ketothiolase deficiency related leukoencephalopathy change.

The patient was managed with aggressive intravenous fluid resuscitation, sodium bicarbonate infusion, correction of electrolyte abnormalities, and metabolic stabilization with high-calorie, glucose-based nutrition and protein restriction. After correction of the acidosis, her neurological status gradually improved. She was discharged on carnitine supplementation, strict dietary protein/isoleucine restriction, and close metabolic follow-up. Interval neuroimaging was planned to monitor basal ganglia and tract-related sequelae.

## Discussion

### Pathophysiology and clinical–imaging correlation

BKTD is a severe inborn error of isoleucine and ketone body catabolism resulting in accumulation of organic acids and recurrent episodes of severe ketoacidosis [1,1]. Mutations in ACAT1 impair mitochondrial acetoacetyl-CoA thiolase, disrupting thiolytic cleavage of 2-methylacetoacetyl-CoA [1]. Neurotoxic metabolites and metabolic acidosis disturb mitochondrial function and oxidative metabolism, particularly in metabolically active deep gray nuclei [1,2,7].

Clinically, BKTD presents with vomiting, poor feeding, seizures, abnormal tone, lethargy, and coma [[1], [2], [3]]. Episodes are typically triggered by infection or fasting. Our patient’s acute encephalopathy closely followed a documented severe ketoacidotic crisis, with previously normal MRI, confirming that the observed brain lesions were acutely acquired.

#### Basal ganglia involvement on CT and MRI

Neuroimaging abnormalities have been reported in a minority of BKTD cases, but when present, deep gray nuclei are preferentially affected. Brismar and Ozand, and Ozand et al. [2] described symmetric T2 hyperintensity and CT hypodensity involving the posterolateral putamina and lentiform nuclei in BKTD, sometimes extending into the external capsule and periventricular white matter [1] These early observations emphasized putaminal involvement.

Later, O’Neill et al. [3] reported a 5-year-old girl with BKTD whose MRI demonstrated isolated bilateral globus pallidus lesions: T2/FLAIR hyperintense with diffusion restriction and no enhancement in the acute phase, evolving after 1 year into nonenhancing T2 hyperintense, T1 hypointense, nonrestricting microcystic cavitary lesions of the globipallidi [2]. Wojcik et al. [4] further described 2 phenotypes: bilateral globus pallidus and substantia nigra involvement with diffusion restriction, and predominant putaminal lesions with scattered white matter abnormalities but without diffusion restriction when imaged subacutely [3].

#### Our case reproduces and extends these patterns

Initial CT showed globus pallidus hypodensity, mirroring the pallidal-predominant pattern described on MRI in O’Neill’s case, but identified on CT due to lack of immediate MRI [2], (Fig. 4). Follow-up CT revealed progression to involve both putamina and the cerebral peduncles, demonstrating that the injury is dynamic and may propagate beyond the pallidi during ongoing metabolic stress (Fig. 5).

Subsequent MRI showed chronic-stage globus pallidus lesions—T2/FLAIR hyperintense, T1 hypointense, nonrestricting, with punctate SWI hypointensities suggestive of microhemorrhage—fully consistent with microcystic cavitary degeneration and irreversible basal ganglia damage described in the chronic phase of BKTD [2,4] (Fig. 6).

#### Extra–basal ganglia and tract involvement

Increasingly, BKTD is recognized to involve extra-basal ganglia structures. Guo et al. [5] reported a 10-month-old girl with severe BKTD whose MRI showed T2/DWI hyperintensities affecting the caudate nuclei, lentiform nuclei, bilateral cerebral peduncles, and mesencephalon (periaqueductal region) [4]. Over time, diffusion restriction resolved and basal ganglia lesions evolved to liquefactive cavitation and atrophy, while brainstem lesions fully regressed [4]. Al-sawadi and Hussain [6] reported combined basal ganglia and cerebellar involvement with multiple cerebellar hypodense foci on CT and T2/FLAIR hyperintensities on MRI that partially regressed but left the patient ataxic [4,5]. Zhang et al. [7] described additional pediatric cases with both basal ganglia and cerebellar abnormalities [5].

In our patient, extra-basal ganglia involvement is clearly demonstrated on CT progression from globus pallidus hypodensity to new hypodensities in the cerebral peduncles, suggesting early tract involvement (Fig. 5). MRI confirm bilateral cerebral peduncle lesions that are T2 hyperintense, T1 hypointense with diffusion restriction, indicating acute/subacute insult (Fig. 6).

Additional nonrestricting T2/FLAIR hyperintense foci in the rightfrontal and right parietal white matter, together with generalized cerebral and cerebellar atrophy, indicate more diffuse brain involvement, likely from repeated or severe metabolic stress (Fig. 7).

This constellation—irreversible pallidal degeneration, acute/subacute peduncular lesions, and widespread white matter abnormalities—is highly concordant with severe BKTD as described by Guo et al. [4] and underscores that BKTD can produce both focal (deep gray and tract) and diffuse (white matter and global atrophy) brain injury.

#### Mechanisms of vulnerability

The basal ganglia, especially the globus pallidus and putamen, have high metabolic demands and are particularly sensitive to fluctuations in oxidative metabolism and pH [1,2,7]. In BKTD, impaired ketone body utilization and accumulation of organic acids lead to energy failure and oxidative stress in these regions [1,7]. The extension of lesions along internal capsules, corticospinal tracts, and cerebral peduncles likely reflects a combination of direct metabolic susceptibility of highly myelinated long tracts and secondary Wallerian degeneration propagated from primary basal ganglia injury [1,4].

Differential diagnosis, the differential diagnosis of symmetric basal ganglia lesions in a child includes other organic acidemias (methylmalonic, propionic, and isovaleric), pyruvate dehydrogenase deficiency, urea-cycle disorders, hypoxic–ischemic injury, carbon monoxide poisoning, Wilson’s disease, and neurodegeneration with brain iron accumulation, among others [[1], [2], [3],^7^]. However, in this case, the known ACAT1-related BKTD, age, severe euglycemic ketoacidosis with normal lactate, highly symmetric nonvascular distribution, and temporally normal prior MRI strongly support BKTD as the etiology. In a child with severe ketoacidosis and acute encephalopathy, bilateral globus pallidus hypodensity on CT should prompt consideration of BKTD and other inborn errors of metabolism. Interval extension of lesions to the putamina and cerebral peduncles indicates ongoing metabolic injury and parallels patterns of tract involvement described on MRI in severe BKTD. MRI demonstrating T2/FLAIR hyperintense, T1 hypointense, nonrestricting globus pallidus lesions with SWI microhemorrhages, plus diffusion-restricting peduncular lesions—helps differentiate irreversible basal ganglia damage from acute tract injury. A previously normal MRI is invaluable for confirming that these findings are acquired and temporally related to the metabolic crisis. Early radiologic recognition should trigger urgent metabolic evaluation and treatment to prevent further irreversible brain injury.

## Conclusion

Beta-ketothiolase deficiency is a rare but important cause of pediatric metabolic encephalopathy with characteristic neuroimaging findings. Our case demonstrates the evolution from isolated globus pallidus hypodensity on CT to more extensive basal ganglia and cerebral peduncle involvement, and MRI evidence of irreversible microcystic cavitary degeneration with microhemorrhage in the globipallidi, acute/subacute peduncular injury, supratentorial white matter involvement, and diffuse cerebral and cerebellar atrophy. Even in resource-limited settings where MRI is not immediately available, serial CT can provide crucial diagnostic and prognostic information. Radiologists play a central role in recognizing these patterns and prompting timely metabolic work-up and management.

## Ethical statement

This type of study does not require any ethical approval by our institution.

## Date availability statement

The authors confirm that the data supporting the findings of this study are available within the article.

## Patient consent

Written informed consent was obtained from the patient’s mother for publication of this case report and accompanying images. A copy of the written consent is available for review by the Editor-in-Chief of this Journal on request.

## CRediT authorship contribution statement

**Berihu Gebre:** Writing – review & editing, Writing – original draft, Visualization, Conceptualization. **Yirgalem Gereziher:** Writing – review & editing, Conceptualization. **Dejen Gebrewahd:** Investigation, Conceptualization. **Catherine Alcober:** Investigation, Conceptualization.
